# Risk Perceptions of Substance Use Recovery Disclosure in Medical School Applications: A National Sample of Physicians and Dentists

**DOI:** 10.1007/s11606-026-10233-9

**Published:** 2026-03-09

**Authors:** Rachel Chang, Nicholas Ganek, James K. Colgrove, Margaret R. Pereyra, Carrigan L. Parish, Petra Jacobs, Viviana E. Horigian, Harold A. Pollack, Daniel J. Feaster, Lisa R. Metsch

**Affiliations:** 1 Department of Sociomedical Sciences, Mailman School of Public Health, Columbia University, New York, NY USA; 2 Columbia College, Columbia University, New York, NY USA; 3 University of Miami Miller School of Medicine, Miami, FL USA; 4 School of General Studies, Columbia University, New York, NY USA; 5 Tulane School of Medicine, New Orleans, LA USA; 6 Department of Sociomedical Sciences, Mailman School of Public Health, Miami Research Center, Columbia University, Miami, FL USA; 7 National Institute of Health, National Institute of Drug Abuse, Bethesda, MD USA; 8 Department of Public Health Sciences, University of Miami Miller School of Medicine, Miami, FL USA; 9 Crown Family School of Social Work, Policy, and Practice, Department of Public Health Sciences, Urban Health Lab, University of Chicago, Chicago, IL USA; 10 Department of Biostatistics, University of Miami Miller School of Medicine, Miami, FL USA; 11 Present Address: National Institute of Health, National Institute on Aging, Bethesda, MD USA

**Keywords:** substance use disorders, provider stigma, medical school admissions, dental school admissions, risk perception

## Abstract

**Aims:**

Given stigmatizing attitudes held by many healthcare providers toward substance use, medical and dental school applicants in recovery from past substance use face the dilemma of whether to disclose this during the admissions process. We assessed current physicians’ and dentists’ perceptions of the risk that such disclosure would have on an applicant’s prospects of admission and how this was influenced if the disclosure was framed as part of a professional journey to help others.

**Design:**

A national probability survey of physicians and dentists using a sampling frame from the American Medical Association and American Dental Associations.

**Setting:**

Online and paper questionnaire.

**Participants:**

One thousand two hundred forty emergency department physicians, primary care physicians, and dentists.

**Measurements:**

A 125-item survey assessing provider stigma toward people with substance use disorders with two questions on the perceived risk of disclosure of substance use on medical and dental school applications.

**Findings:**

A total of 82.5% of physicians and dentists reported that disclosure of a history of substance use by medical or dental school applicants would carry a “moderate” or “significant” risk for adverse admissions outcomes. When past substance use was framed as part of an applicant’s professional motivation, half of the respondents (49.8%) perceived disclosure as “moderately” or “significantly” risky. Respondents who reported having a friend or patient who uses or used drugs were more likely to perceive substance use disclosure (without context) as riskier than their corresponding colleagues.

**Conclusion:**

Physicians and dentists overall perceive high risk for adverse admissions outcomes when applicants disclose their past substance use in their medical and dental school applications. This risk is lessened yet remains prominent when the experience is framed as part of the applicant’s professional motivation. This may reflect a certain persistence of stigma among physicians and dentists toward people with a history of substance use.

**Supplementary Information:**

The online version contains supplementary material available at 10.1007/s11606-026-10233-9.

## INTRODUCTION

Healthcare provider stigma is an increasingly recognized barrier to expanding access to substance use disorder (SUD) care^1^. Although effective treatments exist, most Americans with opioid use disorder remain untreated. Stigma—a process by which people with a specific social identity are labeled, stereotyped, devalued, and discriminated against^2^—contributes to this treatment gap. In healthcare, stigma is particularly important, given the unequal power relations between providers and patients and the specific need to engage patients who are ambivalent in seeking SUD treatment.

Provider stigma toward patients with SUDs can manifest in negative attitudes^3^, compassion fatigue^4^, and therapeutic pessimism^5^, all of which may worsen health outcomes. Furthermore, such stigma has also been linked to inadequate training, which in turn perpetuates stigma as providers may feel unequipped to treat these patients effectively^4^. While didactic education alone has shown limited impact in reducing such stigma, studies have shown that the most effective interventions involve direct, recurrent interactions with individuals with SUD^6,7^. In the context of professional settings, having colleagues or peers with histories of SUDs may be a form of repeated and direct contact.

These dynamics are relevant even in the earliest stages of a medical or dental career. As SUDs are prevalent and increasing among young adults ^24^, many applicants to medical and dental school will likely have such histories. During the medical and dental school application process, aspiring physicians and dentists must decide what information, if any, to share about their recovery. Importantly, medical and dental school applications do not ask about substance use history. However, both the Association of American Medical Colleges (AAMC) and the Associated American Dental Schools (ADEA), which oversee US medical and dental school admissions, respectively, require a personal statement. This essay broadly asks applicants to explain why they wish to attend medical school or pursue a career in oral health; both applications specifically encourage the discussion of “unique hardships, challenges, and obstacles” (AMCAS Section 8) and “experiences, background, and uniqueness”^22^. For applicants in recovery, such prompts offer an opportunity to describe how their experiences shaped their motivation for wanting to pursue a career in medicine or dentistry to help future patients facing similar challenges. Disclosure might occur at other points of the application process, such as during interviews where applicants may be asked about educational gaps or interests in addiction-related fields. Yet, applicants may fear that such honesty could hurt their candidacy and lead to immediate rejection, especially in an already competitive admission process, where fewer than 40% of medical school applicants were accepted in 2022 (AAMC), and only about 60% of first-time dental applicants were accepted and enrolled in 2023 (American Dental Education Association, ADEA).

These concerns about the stigma of disclosure mirror broader patterns in the healthcare field. The SUD rates among physicians are similar to or even higher than those of the general population^8^, with approximately 10–12% of physicians developing substance use in their careers^9^. Yet, the fear of prevailing social stigma and professional repercussions, described as a “conspiracy of silence,” appears to be associated with physicians’ delayed diagnoses and reluctance to seek treatment^10^. This phenomenon may influence the early stages of a potential doctor’s journey even if the applicant is in recovery. To our knowledge, no published empirical study explores how physicians and dentists perceive the risk of disclosing a history of substance use or recovery on medical or dental school admission applications. This manuscript seeks to address this gap in the current literature.

## METHODS

We conducted a national survey of a stratified random sample of emergency department physicians, primary care physicians, and dentists to assess their stigma toward patients with SUDs. The study protocol has been described in detail previously in Parish et al.^11,12^. The sampling frame was provided by RediData, a licensed data provider for the American Medical and American Dental Associations. The University of Miami Institutional Review Board (UM; IRB# IRB00010711) approved this study.

Employing the total design method^13^, the National Opinion Research Center (NORC) administered the survey via email and mail. Participants were sent a pre-notification letter, initial and replacement questionnaires, and reminders via email, telephone, physical mail, and fax. Incentives to boost participation included $10 for survey initiation, $50 for completion, and a bonus $100–250 that was offered to chronic nonrespondents. The response period spanned from October 2020 to October 2022.

The survey questionnaire consisted of 125 questions assessing the clinical scope of physicians and dentists, provider stigma, patient demographics, and provider “regard”^2^. Measures of provider stigma were adapted from studies assessing mental health stigma and focused on perceived dangerousness of participants, subjective norms, and social desirability regarding treating patients with SUDs. Provider stigma was assessed through Medical Condition Regard Scales (MCRS), 11-item scales gauging providers’ attitudes toward the enjoyment, treatability, and worthiness of caring for patients with specific conditions^14^. These medical conditions included three SUD categories (opioid use, stimulant use, and alcohol use) and three chronic conditions (type II diabetes mellitus, depression, and HIV). Surveys were confidential with data linked to non-identifying study identification numbers.

Two questions gauged the providers’ views on medical or dental school admissions concerning a potential applicant’s history of substance use and recovery (see Appendix). Respondents assessed the risk associated with two hypothetical applicants, both contemplating disclosing their history of substance use recovery in their application. Applicant A was described as having “a history of drug or harmful alcohol use. He did not have a gap in his education or work history and did not have any institutional/criminal justice sanctions.” In the other scenario, Applicant B had the same background but wanted “to enter the field of addiction psychiatry” for medical school or “the intersection of oral health and substance use” for dental school where “his past drug or harmful alcohol use and recovery history is part of his professional motivation and drive to help others.” For each scenario, survey respondents were instructed to rate the potential risk of such a disclosure on the applicant’s admission prospects as having “No Risk,” “Low Risk,” “Moderate Risk,” or “Significant Risk.”

As is well established in stigma research, we measured perceptions of others’ attitudes (perceived public stigma) rather than respondents’ personal attitudes, since disclosure decisions are often guided by what individuals believe others will think^15^.

### Measures

The dependent variables used in this analysis were the presence of risk for disclosure on medical or dental school applications under the two scenarios. Risk was dichotomized as “significant”/“moderate” risk versus “low”/“no risk” in order to assess the presence of risk under each scenario. Basic sociodemographic characteristics of providers were included in the analysis as independent variables (including gender, race/ethnicity, and age). Other covariates included the degree of stigmatizing attitudes toward patients who use drugs, familiarity with family, friends, patients, or physician colleagues who use drugs or are in recovery, the recency of training in treating SUDs, specialty in medicine, service on a medical school admissions committee, and background and perceived efficacy in treating patients who use drugs.

### Data Analysis and Statistical Methods

Associations between each independent variable and degree of risk for each scenario were assessed using odds ratios for continuous independent variables and cross-tabulations for categorical independent variables. We then used multivariable logistic regression models to examine associations between independent variables and the degree of risk for each scenario. Independent variables significant at the *p* = 0.25 level in bivariate analyses, along with a set of relevant sociodemographic variables (age, gender, urbanicity, race, and provider type), were included in initial models to allow for the detection of potentially relevant covariates and confounding factors. Each initial model was reduced so only covariates with *p* < 0.05 and demographics were retained. This analysis used listwise deletion, where any cases with missing data on the variables of interest were excluded. Analyses were conducted using Stata 18.0 software (Stata Corp 2023). All statistical tests were two-sided, and *p*-values < 0.05 were considered statistically significant. Inverse-probability weights were calculated from the population totals and adjusted for non-response.

## RESULTS

### Sample Characteristics

The final sample size of responders (*N* = 1240) consisted of 349 emergency medicine, 506 primary care physicians (PCPs), and 385 dentists. Out of the initial sampling frame of *N* = 3011, there was a Council of American Survey Research Organizations (CASRO) survey response rate of 53.6%, calculated after excluding clinicians who were ineligible due to retirement or withdrawal from the workforce. There was a higher CASRO response rate among dentists (61.4%) and ER physicians (58.2%). Incentive amount was not associated with any provider characteristics.

The majority (74.2%) of the 1240 respondents identified as white, and 61.3% identified as male (Table 1). The median age of the sample was 51 years old. The median year that respondents received an MD, DO, DDS, or DMD degree was 1997, indicating that they had been practicing for approximately 23 years at the time of survey administration. Around half of respondents practiced in a private setting (53.2%), with 26.5% in solo private practice and 26.7% in group private practice. Two-thirds of respondents accepted Medicaid (62.8%), and 72.6% of respondents reported completing a continuing medical/dental education course on treating drug use. Notably, 13.4% reported having served on an admissions committee for a medical school or a graduate medical education or dental school.

**Table 1:** Sample Characteristics of the Physicians and Dentists Who Participated in This Study. Note: Percentages are Weighted

	*N*	Percentages
**Gender**		
Male	818	61.3%
Female	408	38.3%
Non-binary/third gender	3	0.4%
**Hispanic or Latino**		
Yes	62	5.5%
No	1162	94.5%
**Race and ethnicity**		
White	949	74.2%
Black or African American	37	3.5%
Asian or Pacific Islander	180	17.7%
Native American or Alaskan Native	3	0.2%
Of mixed racial background	18	1.6%
Some other race	30	3.0%

Table 2 shows that for Applicant A (without contextualization), 82.5% rated substance use disclosure as either “moderately” or “significantly” risky (with 38.0% indicating “significant” risk, 44.5% “moderate risk,” 15.6% “low risk,” 2.0% “no risk”). In the case of Applicant B, when the substance use history was contextualized as part of the applicant’s motivation, 49.8% of the respondents still perceived disclosure as either “moderately” or “significantly” risky (with 12.2% indicating “significant risk,” 37.6% with “moderate risk,” 42.2% “low risk,” and 8.0% “no risk”).

**Table 2:** Frequency and Risk Assessment of Disclosure in Hypothetical Scenarios for Applicant A and Applicant B by Physicians and Dentists

	Applicant A scenario		Applicant B scenario	
Degree of risk of disclosing	Count	Percentages	Count	Percentages
No risk	25	2.3%	99	8.0%
Low risk	193	15.9%	525	42.4%
Moderate risk	552	45.8%	467	37.8%
Significant risk	471	36.0%	152	11.9%
**Total**	1241	100.0%	1243	100.0%

### Variables Associated with Physician’s or Dentist’s Risk Perception of Disclosure Risk

For Applicant A, physicians and dentists who reported having a friend who uses drugs or is in recovery were more likely to perceive substance use disclosure as “moderately” or “significantly” risky than those without such friends (84.1% vs 78.5%). White physicians and dentists were more likely to perceive Applicant A’s disclosure as “moderately” or “significantly” risky than providers of other races (84.0% vs 77.1%). There was no significant difference in risk perceptions among respondents who had and had not previously served on a medical or dental school admissions committee (see Appendix for Supplemental Table of more univariate associations).

### Multivariable Regression Models of Physicians’ and Dentists’ Perception of Risk in Disclosing Substance Use History in Medical or Dental School Application

This analysis revealed associations influencing the perceived risk of substance use disclosure for medical or dental school applicants (Table 3). For Applicant A, physicians and dentists with positive attitudes toward individuals actively using drugs were less likely to report disclosure as “moderately” or “significantly” risky (OR = 0.57, CI [0.41, 0.81]). Those with a friend using drugs or in recovery were more likely to perceive it as “moderately” or “significantly” risky (OR = 2.25, CI [1.24, 4.08]). White physicians (OR = 1.48, CI [1.03, 2.13]) and those who had treated individuals with substance use or in recovery (OR = 1.44, CI [1.03, 2.00]) showed an increased likelihood of considering Applicant A’s disclosure as “moderately” or “significantly” risky. Physicians and dentists exhibiting higher stigma toward alcohol use, as indicated by MCRS alcohol scores, were associated with an elevated perception of risk for Applicant A’s disclosure (OR = 1.24, CI [1.06–1.47]).

Different variables were associated with the risk for Applicant B. Physicians and dentists with positive attitudes toward individuals in recovery perceived less risk than those with negative attitudes (OR = 0.61, CI [0.49, 0.77]). Confidence in seeking help from others for consulting purposes (see Appendix) was associated with a decreased likelihood of perceiving Applicant B’s disclosure as “moderately” or “significantly” risky (OR = 0.83, CI [0.69, 0.99]). Conversely, physicians and dentists who believed others had negative beliefs about people who use drugs (OR = 1.27, CI [1.06, 1.54]) and those confident in their ability to help patients who used drugs (OR = 1.45, CI [1.13, 1.86]) were more likely to consider Applicant B’s disclosure as moderate or significantly risky.

**Table 3:** Multivariate Analysis of Factors Influencing Odds of Reporting Disclosure Risk* in Applicant A and Applicant B Scenarios

Variable	Applicant A scenario			Applicant B scenario		
	**Odds ratio A**	**95% CI** **A**	***p*-value A**	**Odds ratio B**	**95% CI** **B**	***p*-value** **B**
Age	1.043	0.885–1.229	0.614	1.036	0.914–1.174	0.614
Male	0.773	0.540–1.108	0.161	0.908	0.693–1.188	0.480
Urban	0.875	0.586–1.305	0.512	1.102	0.819–1.484	0.520
White race vs all others	**1.482**	1.032–2.129	0.033	1.000	0.741–1.349	1.000
Provider type (MD)	1.000	--	--	1.000	--	--
Provider type (ER)	1.103	0.734–1.657	0.638	1.032	0.760–1.401	0.842
Provider type (Dentist)	0.885	0.608–1.288	0.523	1.147	0.853–1.543	0.364
Positive attitude toward people actively using drugs	**0.571**	0.405–0.806	0.001	--	--	--
Has a friend who uses or in recovery	**2.249**	1.240–4.080	0.008	--	--	--
Has treated someone who actively uses drugs or is in recovery	**1.437**	1.031–2.002	0.032	--	--	--
Medical Condition Regard Scale (MCRS) for alcohol	**1.246**	1.056–1.471	0.009	--	--	--
Believes others have negative beliefs	--	--	--	**1.274**	1.055–1.539	0.012
Has a positive attitude toward persons in recovery	--	--	--	**0.612**	0.487–0.770	0.000
Confident in consulting others	--	--	--	**0.825**	0.691–0.985	0.033
Confident in the ability to help and treat patients who use drugs	--	--	--	**1.448**	1.126–1.862	0.004

*Disclosure risk is grouped as “significant” or “moderate” versus “low” or “no risk”

Scenarios A and B were modeled separately. “--” refers to variables not retained in the final model for each scenario. See “METHODS”

## DISCUSSION

This study examined physicians’ and dentists’ perceptions of the risk of substance use disclosure by medical and dental school applicants. In addition to assessing how the respondents themselves felt about the potential risk of disclosure, our study examined the respondents’ perception of their colleagues and the prevailing culture within their fields. The providers’ perceptions are therefore a valuable indicator of both the extent of stigma in healthcare toward people who use drugs and potential barriers facing aspiring physicians and dentists with a history of substance use. Three primary findings emerge from this study.

Firstly, there is a widespread perception of high risk associated with disclosing past substance use in medical or dental school admissions. Most physicians and dentists agreed that disclosing a history of substance use would be either “moderately” or “significantly” risky to an applicant’s admission prospects. Even when framed within an intellectual and professional arc, the perceived risk remains, indicating an overarching hesitancy toward such disclosures.

Secondly, additional context that frames the applicant’s substance use recovery as integral to their professional motivation has some association with perceived risk in admission prospects. While both scenarios of disclosure were viewed with high risk, there was a discernible decrease in risk perception for Applicant B. This finding suggests that how applicants explain their history may influence the way it is perceived. The question posed about Applicant B was designed to mirror the experience of applicants who respond to application prompts like “Why Medicine?” or “Why Dentistry?” by detailing personal growth amid adversity with substance use. As the AAMC explicitly encourages applicants to address “unique hardships, challenges, and obstacles that may have influenced their educational pursuits,” it is reasonable that an applicant who has recovered from substance use would choose to share those experiences, particularly if that is why they chose to pursue medicine (AAMC, Personal Comments Essay). With the rising prevalence of substance use among young adults and the general US population, more applicants may share similar experiences in recovery and aspire to practice medicine to aid others facing similar challenges. However, this data indicates that even with such context, sharing these experiences may be a considerable risk to the applicant’s chances.

The third key finding is the association between the providers’ familiarity with individuals with SUDs and their perception of disclosure risk, with the direction of the association varying by the nature of that familiarity. Across both applicants (A & B), self-reported positive attitudes toward people who use drugs or are in recovery were associated with lower perceived risk, whereas more direct experiences—such as treating individuals with SUDs or expressing confidence in providing such care—were associated with higher perceived risk. Although confidence in providing care does not necessarily capture actual treatment exposure, these findings indicate that different forms of familiarity may relate to risk perception in distinct ways. Additional research is needed to clarify these relationships; for instance, more direct contact may correspond to greater awareness of potential discrimination, while positive attitudes may simply reflect attitudinal acceptance.

Independent of general attitudes, physicians’ or dentists’ personal experiences significantly affect their perception of disclosure risk for both Applicant A and Applicant B. Providers who reported having a friend or family member with SUD or a history were associated with a greater perceived risk compared to professional interactions with patients. These personal connections may offer deeper insights into the social challenges and stigmatization faced by individuals with substance use, potentially heightening providers’ sensitivity to the risks of disclosing. This finding aligns with previous studies that found that direct, recurrent interactions with individuals with substance use are the most promising avenues for mitigating physician stigma (Livingston 2012; Van Brakel 2019). This process of reducing stigma inherently involves cultivating empathy and understanding, requiring an informed awareness of the challenges and discrimination encountered by these individuals.

Interestingly, age was not a significant factor to risk perception. If recent medical training had reduced stigma, younger respondents might have shown lower risk than older ones. This absence of difference suggests that attention to stigma reduction in medical and dental training may remain limited. Our finding that white health providers were more likely to perceive both applicants as risky raises questions on the role of the provider. This difference may reflect a difference in cultural attitudes or varying exposures to communities affected by SUD. Future research could explore race's mechanisms of how provider race may influence perceptions of risk.

It is also notable that 11.2% of respondents reported knowing a physician/dentist colleague who actively uses drugs and 31% reported knowing a colleague who is in recovery, which is consistent with existing literature of higher substance use in physicians.

Our findings align with those reported by Gold and colleagues^16^ who examined substance use disclosure among applicants to medical licensure. They found that while 47 states asked about substance use on their licensing application, only 8 states specifically asked about current use. While their study highlighted how structural elements such as broad health questionnaires discourage physicians from seeking help, our study provides complementary evidence by assessing physicians’ perceptions of risk when applicants disclose substance use during the admissions process. In a review of related studies, Taylor and colleagues^17^ advocated for increased transparency through faculty disclosures of SUD recovery, suggesting that such efforts could normalize and foster a culture in academic medicine where healthcare professionals may be more willing to address not only their own well-being but also that of their patients.

Our results have some limitations. Our cross-sectional data constrain our ability to establish causation. While comparing the risk perception of Applicant A and Applicant B provides a nuanced analysis of stigma, we do not directly ask respondents if they would personally admit such applicants, as this would likely require more information about the applicants (e.g., GPA, MCAT scores, clinical experience). The absence of this question limits our ability to directly compare personal stigma with perceived stigma, introducing uncertainty in comparing how physicians’ own views align with their expectations of colleagues. For instance, a respondent may value the unique experiences in a recovered applicant and personally support their acceptance yet still view disclosure as risky due to institutional norms or a broader culture of limited acceptance in medicine and dentistry. Our approach, centered on perceived risk and stigma, allowed us to explore how professional attitudes might influence the evaluation of applicants who disclose a history of substance use. Further investigations could include questions directly asking if the physician would personally admit such applicants to assess how personal and perceived stigma each shape disclosure.

Another limitation is the lack of differentiation among types of substance use, or between drug use and alcohol use. We asked about “harmful drug or alcohol use,” without specifying the drug as marijuana, stimulants, or opioids, which could have influenced respondents’ attitudes differently and potentially revealed distinct perspectives on these substances. Likewise, alcohol’s legality and widespread social acceptability could shape how applicants perceive and disclose their alcohol use differently from illicit drugs. Future research could explore how different substance use types might influence the perceived risk in medical and dental school admissions.

Our sample population also poses limitations to the generalization of our findings, as it included PCPs (inclusive of specialties of internal medicine, family medicine, general medicine, pediatrics, and obstetrics), emergency physicians, and dentists, but excluded surgeons and subspecialty procedural specialists. Because relapse in highly procedural fields may pose greater risks to patient safety, these health providers might hold different perceptions of applicants with a history of SUD.

Lastly, the applicant scenarios were consistently presented in the same order, with Applicant A preceding Applicant B. This sequence may have biased perceptions by encouraging direct comparisons, potentially making Applicant B appear more favorable relative to Applicant A. Future research could randomize case order to test such effects. In addition, between the physician and dentist surveys, differences in survey wording may have influenced responses, as “addiction medicine” is a recognized medical specialty, whereas there is no equivalent “addiction” specialty that exists within dentistry.

Despite these limitations, this study is the first to report on the high perceptions of risk associated with disclosing a history of substance use recovery in medical and dental school applications, drawing on a national probability sample of physicians and dentists. Given the current overdose crisis and the rising substance use rates in the USA, there is a need for innovative measures to better understand the prevalent stigma that deters people with SUDs from fully integrating into society. Understanding the barriers faced by aspiring doctors and dentists in recovery from substance use when applying to schools may help shift stigmatizing attitudes in healthcare and better respond to this public health priority.

Overall, the risk perceptions reported in this study reflect the ongoing challenges of stigma toward substance use within the medical field. While this research focuses on the admissions process, its findings point to broader implications regarding how stigma is addressed among healthcare professionals. These efforts may be essential for building an inclusive medical and dental care workforce and providing proficient and humane care to all persons who experience SUDs.

## Supplementary Information

Below is the link to the electronic supplementary material.

Supplementary file1 (DOCX 21.4 KB)

Supplementary file2 (DOCX 477 KB)

Supplementary file3 (DOCX 149 KB)

## Declarations

This article was initiated while Dr. Petra Jacobs was employed at the National Institute on Drug Abuse. The opinions expressed in this article are the author's own and do not reflect the view of the National Institutes of Health, the Department of Health and Human Services, or the United States government.
