# Supplementary material for: Risk Perceptions of Substance Use Recovery Disclosure in Medical School Applications: A National Sample of Physicians and Dentists
Source: J Gen Intern Med. 2026 Mar 9;41(9):2560–6. doi: 10.1007/s11606-026-10233-9 (PMC13304001; doi:10.1007/s11606-026-10233-9)
Supplement: Supplementary file 1 — Supplementary file1 (DOCX 21.4 KB) [file 11606_2026_10233_MOESM1_ESM.docx]

| **SUPPLEMENTAL TABLE: Disclosure Risk* Perception by Provider Characteristics - Applicant A and Applicant B Scenarios**  *Disclosure risk is grouped as “significant” or “moderate” versus “low” or “no risk”.   \|  \|  \| **Applicant A** \| \| \| **Applicant B** \| \| \| \| --- \| --- \| --- \| --- \| --- \| --- \| --- \| --- \| \| **Independent Variable** \| **Response** \| **Disclosure risk % *** \| **N** \| **p-value** \| **Disclosure risk % *** \| **N** \| **p-value** \| \| Has completed Continuing Medical/Dental Education courses in treating drug use \|  \|  \|  \| 0.133 \|  \|  \| 0.389 \| \| no \| 85.4% \| 328 \|  \| 52.4% \| 330 \|  \| \| yes-within past year \| 82.1% \| 485 \|  \| 48.0% \| 485 \|  \| \| yes-more than 1 year ago \| 79.6% \| 382 \|  \| 47.9% \| 382 \|  \| \| Emergency Department \|  \|  \|  \| 0.362 \|  \|  \| 0.942 \| \| no \| 81.8% \| 874 \|  \| 49.7% \| 876 \|  \| \| yes \| 84.0% \| 350 \|  \| 49.4% \| 350 \|  \| \| Has served on medical/dental school or graduate medical/dental education admissions committee \|  \|  \|  \| 0.874 \|  \|  \| 0.307 \| \| no \| 82.4% \| 1,053 \|  \| 50.1% \| 1,055 \|  \| \| yes \| 81.9% \| 166 \|  \| 45.8% \| 166 \|  \| \| Knows a physician/dentist colleague who is in recovery for using drugs \|  \|  \|  \| 0.616 \|  \|  \| 0.776 \| \| no \| 81.9% \| 830 \|  \| 49.7% \| 831 \|  \| \| yes \| 83.1% \| 379 \|  \| 48.8% \| 381 \|  \| \| Has a friend who uses or is in recovery \|  \|  \|  \| 0.015 \|  \|  \| 0.464 \| \| no \| 78.5% \| 409 \|  \| 50.9% \| 409 \|  \| \| yes \| 84.1% \| 801 \|  \| 48.6% \| 804 \|  \| \| Has a relative who uses or is in recovery \|  \|  \|  \| 0.84 \|  \|  \| 0.342 \| \| no \| 82.5% \| 514 \|  \| 51.0% \| 514 \|  \| \| yes \| 82.0% \| 696 \|  \| 48.20% \| 699 \|  \| \| Has treated someone who uses or is in recovery \|  \|  \|  \| 0.429 \|  \|  \| 0.439 \| \| no \| 81.1% \| 472 \|  \| 48.0% \| 473 \|  \| \| yes \| 82.9% \| 738 \|  \| 50.3% \| 740 \|  \| \| Familiarity with colleagues who use drugs \|  \|  \|  \| 0.539 \|  \|  \| 0.838 \| \| no \| 81.7% \| 782 \|  \| 49.6% \| 783 \|  \| \| yes \| 83.1% \| 421 \|  \| 48.9% \| 423 \|  \| \| Male \|  \|  \|  \| 0.173 \|  \|  \| 0.79 \| \| no \| 84.5% \| 419 \|  \| 50.1% \| 419 \|  \| \| yes \| 81.4% \| 805 \|  \| 49.3% \| 807 \|  \| \| White race \|  \|  \|  \| 0.01 \|  \|  \| 0.579 \| \| no \| 77.1% \| 262 \|  \| 50.8% \| 262 \|  \| \| yes \| 84.0% \| 935 \|  \| 48.8% \| 936 \|  \| \| Urban \|  \|  \|  \| 0.394 \|  \|  \| 0.621 \| \| no \| 84.3% \| 248 \|  \| 48.2% \| 249 \|  \| \| yes \| 82.0% \| 976 \|  \| 50.0% \| 977 \|  \| \| Provider type \|  \|  \|  \| 0.601 \|  \|  \| 0.273 \| \| MD \| 82.3% \| 497 \|  \| 47.3% \| 499 \|  \| \| ER \| 84.0% \| 350 \|  \| 49.4% \| 350 \|  \| \| Dentist \| 81.2% \| 377 \|  \| 52.8% \| 377 \|  \| |
| --- | --- | --- | --- | --- | --- | --- | --- | --- | --- | --- | --- | --- | --- | --- | --- | --- | --- | --- | --- | --- | --- | --- | --- | --- | --- | --- | --- | --- | --- | --- | --- | --- | --- | --- | --- | --- | --- | --- | --- | --- | --- | --- | --- | --- | --- | --- | --- | --- | --- | --- | --- | --- | --- | --- | --- | --- | --- | --- | --- | --- | --- | --- | --- | --- | --- | --- | --- | --- | --- | --- | --- | --- | --- | --- | --- | --- | --- | --- | --- | --- | --- | --- | --- | --- | --- | --- | --- | --- | --- | --- | --- | --- | --- | --- | --- | --- | --- | --- | --- | --- | --- | --- | --- | --- | --- | --- | --- | --- | --- | --- | --- | --- | --- | --- | --- | --- | --- | --- | --- | --- | --- | --- | --- | --- | --- | --- | --- | --- | --- | --- | --- | --- | --- | --- | --- | --- | --- | --- | --- | --- | --- | --- | --- | --- | --- | --- | --- | --- | --- | --- | --- | --- | --- | --- | --- | --- | --- | --- | --- | --- | --- | --- | --- | --- | --- | --- | --- | --- | --- | --- | --- | --- | --- | --- | --- | --- | --- | --- | --- | --- | --- | --- | --- | --- | --- | --- | --- | --- | --- | --- | --- | --- | --- | --- | --- | --- | --- | --- | --- | --- | --- | --- | --- | --- | --- | --- | --- | --- | --- | --- | --- | --- | --- | --- | --- | --- | --- | --- | --- | --- | --- | --- | --- | --- | --- | --- | --- | --- | --- | --- | --- | --- | --- | --- | --- | --- | --- | --- | --- | --- | --- | --- | --- | --- | --- | --- | --- | --- | --- | --- | --- | --- | --- | --- | --- | --- | --- | --- | --- | --- | --- | --- | --- | --- | --- | --- | --- | --- | --- | --- | --- | --- | --- | --- | --- | --- | --- | --- | --- | --- | --- | --- | --- | --- | --- | --- | --- | --- | --- | --- | --- | --- | --- | --- |
