# Supplementary material for: Risk Perceptions of Substance Use Recovery Disclosure in Medical School Applications: A National Sample of Physicians and Dentists
Source: J Gen Intern Med. 2026 Mar 9;41(9):2560–6. doi: 10.1007/s11606-026-10233-9 (PMC13304001; doi:10.1007/s11606-026-10233-9)
Supplement: Supplementary file 2 — Supplementary file2 (DOCX 477 KB) [file 11606_2026_10233_MOESM2_ESM.docx]

**APPENDIX**

**Figure 1:** Questions about Applicants A (without context) and B (with context) in Physician and Dentist Surveys

|  | Applicant A (without context) | Applicant B (with context of professional motivation and drive to help others) |
| --- | --- | --- |
| Physician survey Questions (#26A and B) | 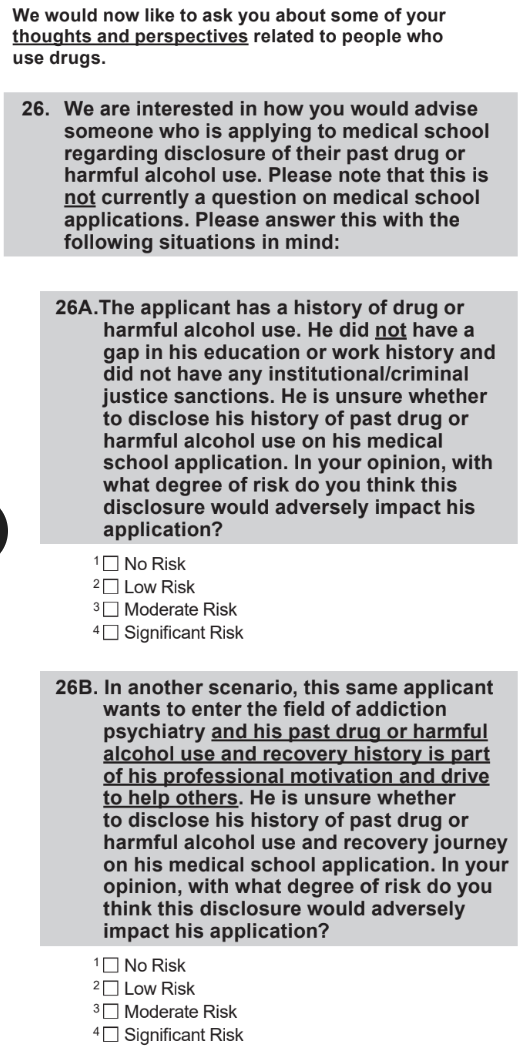 | 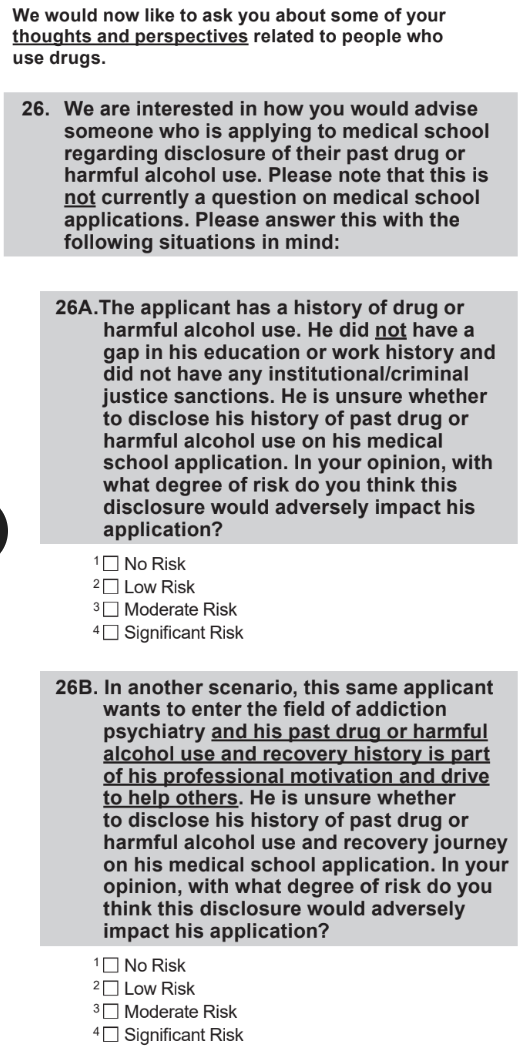 |
| Dentist Survey Questions (#24 A and B | 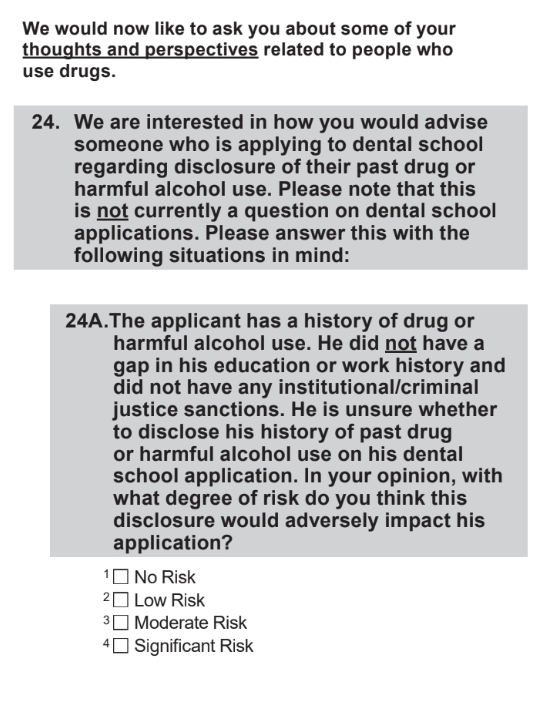 | 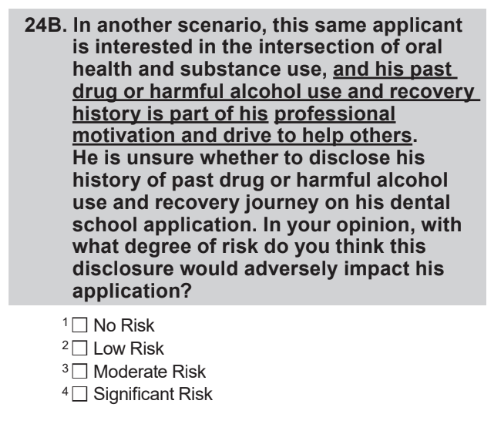 |
