# Supplementary figures and images for: Risk Perceptions of Substance Use Recovery Disclosure in Medical School Applications: A National Sample of Physicians and Dentists
Source: J Gen Intern Med. 2026 Mar 9;41(9):2560–6. doi: 10.1007/s11606-026-10233-9 (PMC13304001; doi:10.1007/s11606-026-10233-9)

**APPENDIX**

**Figure 2**: Question that measured confidence in seeking help from others

| 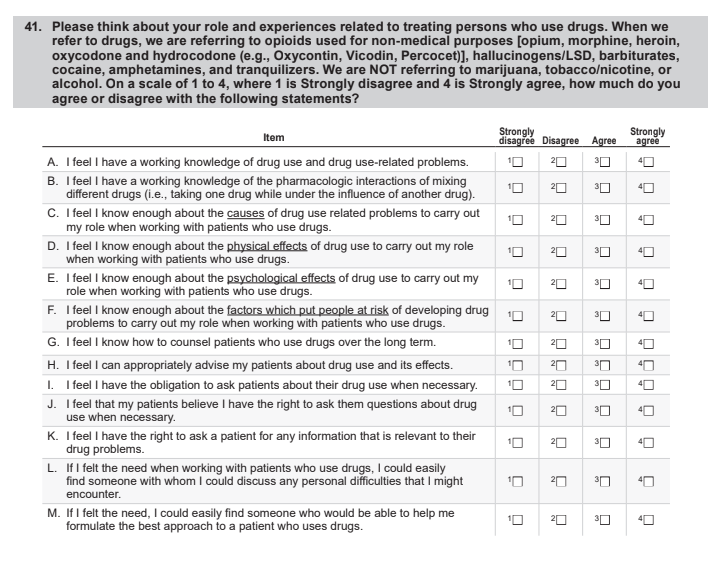 |
| --- |

Supplement: Supplementary file 3 — Supplementary file3 (DOCX 149 KB) [file 11606_2026_10233_MOESM3_ESM.docx]
